# Fourth Dentition

**Published:** 1848-01

**Authors:** 


*Fourth Dentition.*—Early in the spring of 1844, Sherman Bartholomew, a young lad, aged fourteen years, called at my office with his mother, for the purpose of having his teeth examined, they both at that time having always resided at our place.

We were doubly surprised on looking at his mouth; first, that he had any superior frontal incisores at all, for we had known of his losing his two superior *permanent* frontals, by a severe fall some four or five years previous; and secondly, to find the vacancy supplied by what appeared to be two *temporary* incisor teeth. All the rest of the teeth were perfect, the whole second dentition being otherwise complete, except the *sapientiœs.*

Perhaps it may be as well at this time to state that the young man shed his temporary frontal teeth as usual, at about seven or eight years; these were soon succeeded by large and well formed permanent ones. About a year and a half afterwards he fell headlong from a high flow of water, near his parent's residence, into a pile of stones, which resulted in completely separating the two frontal incisores from their sockets, and driving them out of his mouth, they were placed back again and every effort made to induce them to remain and heal, but to no avail, and they were removed, (these the young man had in his possession at the time.)

Thus time passed on, the remaining permanent teeth gradually taking their respective places in the jaw; the two lateral superior incisores continually approaching each other, apparently with the *intention* of filling up the interval between them so far as possible, until about two years after the accident referred to, two frontal *temporary* incisores made their appearance and dropped down between the permanent laterals, just filling the space, as regards *breadth,* though shorter as compared with their neighbors in length. These *triplets* he wore some three years or more, when he came to my office as referred to.

We found a large dual-shaped bunch immediately above the specified teeth, directly under the upper lip, and found, also, the two frontal teeth quite loose. We coincided with the mother in the opinion that teeth were concealed beneath the bunch, and only awaited a favorable opportunity to make their appearance, We extracted the second temporary set; found no roots to them, they having been previously absorbed. We directed the young man to call at our office about a week from that time' he did so, the teeth within the bunches had apparently extended considerably downwards, and the gums *seemed* in a proper condition to lance, they being whitened and evidently constrained over the points of the teeth within the jaw. On lancing them my anticipations were realized in finding teeth immediately under the places referred to.

In a few weeks two large permanent incisores made their appearance; as they became more and more visible, we made efforts to direct them within the circle of the jaw to take the place of the former ones. To this end, finding one of them directly over the right lateral incisor, and also, owing to there not being sufficient room for the "new comers" between the lateral incisores, we extracted the right lateral incisor. The right frontal incisor soon found its proper place; but we found great difficulty in changing the position of the left frontal, indeed we could not move it at all from its original place, though we adopted the usual means and continued them for several months, we were at quite a loss to account for this, until our attention was again directed to a small protuberance within the circle of the jaw, and immediately under the untractable tooth, and evidently preventing it from coming down to its place. We at once conceived the idea that another tooth made its home within the protuberance, and that it soon would emenate from its place of concealment, and show its *classification* and *order!* We had come to the conclusion that after such a profusion of incisores, nature true to her "fondness for variety" would give us a tooth of a different character, but whether it would be molar, bicuspid or cuspidati, the future was to show. We did not, however, wait long to have our curiosity gratified, in a short time the tooth presented itself from the protuberance within the dental arch. This last, was of no particular *order*, indeed altogether "out of order!" a kind of *hybrid*, something between an incisor and cuspidatus. This we extracted, when the left incisor soon came down to its place.

A few months afterwards, the patient, with his parents, removed to the state of Illinois. During the past summer one of my neighbors received a letter from the mother of my patient, in which was a message to us, to the effect that her son had had another tooth within the circle of the jaw under the *right* frontal incisor, corresponding in position with the one we last extracted, on the opposite side; and that on extracting it, they found it corresponded in every respect, so far as they could recollect, with the one we extracted more than three years before.

There can be no doubt of the genuineness of this strange "freak of nature." The first *permanent* set of frontals we saw in the mouth of the patient shortly after the accident which resulted in their loss, and we have also seen them subsequently. The second *temporary* set we extracted ourselves. The *second* permanent set, or *fourth* set proper, are in the head of the patient at this time. One of the *fifth* succession we also extracted. Relying with confidence, as we do, upon the authority of the mother, together with the analogy of the case, we have no doubt of the *sixth* presentation.

The fourth incisores are decided and distinct in their character, only differing from ordinary teeth in that the outline of their lower edge is somewhat unusually undulating or dentated; they are well formed and of good texture and color, leaving an uncertainty as to their true classification; and the conclusion is irresistible that they constitute a genuine case of *fourth dentition!* The two others which followed at intervals, afterwards, were probably nothing more than supernumeraries.

Taking in view the age of the patient, the rapid reproduction and succession of the teeth, their good texture and quality, we consider it one of the most remarkable cases that ever came under our observation, or that we have ever seen on record. We will not at this time enter the field of speculation to attempt to account for the principles by which this singular manifestation of nature was produced, having already extended this report too far.

A box containing representations in wax of the case at *four* different stages of progress, together with one of the second temporary teeth, and one of the supernumerary ones, is in the Museum of the Baltimore Dental College.—*Cazenovia Ed.*

[The above report was intended by the writer to be illustrated with engravings, but it came to hand at so late an hour, that it was impossible to have them made in time for the present number; consequently, we were compelled to omit them. The models of the case are beautifully prepared and arranged, and may be seen at any time in the Museum of the Baltimore College of Dental Surgery.—*Balt. Ed.*]

